# Effect of Recycled Polyvinyl Butyral (rPVB) Addition on the Tribological Performance of Glass–Fiber Reinforced Polyamide (PAGF) during Reciprocating Sliding Wear Conditions

**DOI:** 10.3390/polym15112580

**Published:** 2023-06-05

**Authors:** Isabel Ariadna Carmona-Cervantes, Iván Campos-Silva, Ulises Figueroa-López, Andrea Guevara-Morales

**Affiliations:** 1 Instituto Politécnico Nacional, Grupo Ingeniería de Superficies, SEPI-ESIME Zacatenco, Mexico City 07738, Mexico; 2 Tecnologico de Monterrey, Escuela de Ingeniería y Ciencias, Atizapán de Zaragoza 52926, Mexico

**Keywords:** recycled PVB, glass–fiber reinforced polyamide, solid lubricant, friction, wear resistance, reciprocating sliding

## Abstract

Plastic recycling in the automotive industry is a priority. In this study, the effect of adding recycled polyvinyl butyral (rPVB) from automotive windshields on the coefficient of friction (CoF) and specific wear rate (*k*) of a glass–fiber reinforced polyamide (PAGF) is investigated. It was found that, at 15 and 20 wt.% of rPVB, it acts as a solid lubricant, reducing CoF and *k* up to 27% and 70%, respectively. Microscopical analysis of the wear tracks showed that rPVB spreads over the worn tracks, forming a lubricant layer, which protects the fibers from damage. However, at lower rPVB content, fiber damage cannot be prevented as the protective lubricant layer is not formed.

## 1. Introduction

Nowadays, plastics are one of the most used engineering materials. Unfortunately, its uncontrolled use and unconscious disposal have led to an increased generation and accumulation of solid waste. The actual global environmental situation makes it necessary to use plastics in a more efficient way. One of the industries that have benefited from the increasing use of plastics is the automotive one, as plastics have allowed the designing and manufacture of lighter-weight, fuel-efficient vehicles [1]. Due to the environmental problems associated with plastics, the recycling, recovery, and reuse of end-of-life vehicles (ELVs) have become a priority. However, plastic recycling in the automotive industry is still complex and challenging due to the large number of resins and other materials used, their heterogeneity, mutual entanglement, and the presence of coatings, composites, and different additives [2].

An example of this is the recycling of polyvinyl butyral (PVB), a clear, tough, and flexible engineering thermoplastic with good adhesion to many surfaces that is widely used in laminated safety glass, mainly for automotive applications. Laminated glass consists of a PVB interlayer sandwiched between glass sheets. At the end of a vehicle’s useful life, approximately 1 kg of PVB sheet could be recycled from its windshield [3,4]. Previous studies [3,4,5] have focused on the recycling of laminated glass, especially on the effective separation of glass from the PVB film. Although it has been reported [6] that recycled PVB (rPVB) still possesses excellent mechanical properties and processing performance, there is still a large volume of rPVB that has not yet been widely destined for recycling [7].

To this date, most of the research on rPVB has focused on the development of super-toughened materials. Polyamide (PA) and polypropylene (PP) have been reinforced with rPVB, and a dramatic increase in their toughness has been found [6,7,8,9]. Valera et al. [6] reported that adding 40 wt.% of rPVB to a PA6 matrix increased its impact strength up to 1400%. Wei et al. [7,8] investigated the performance of PA/rPVB blends and the effect of using poly(ethylene-1-octene) (POE-g-MA) as a compatibilizer. They obtained PA/rPVB/POE-g-MA blends with impact strengths 24 times higher than pure PA. Zanjanijam et al. [9] reported that rPVB could act as an elastomer and improve the impact resistance of brittle polymers such as PP during Izod impact tests.

In this study, another use for rPVB is suggested based on its good adhesion and low shear strength: solid lubricant for glass fiber reinforced PA (PAGF). PA is characterized by its high strength, excellent corrosion resistance, and acceptable wear resistance, and when reinforced with glass fibers, its friction and wear performance are improved. However, further enhancements are still required to meet more demanding tribological requirements, and one way of doing so is by using internal lubricants such as polytetrafluoroethylene (PTFE), graphite, and ultrahigh molecular weight polyethylene (UHMWPE) [10,11] that further reduce its wear rate and/or friction coefficient.

Previous work by Verma et al. [12,13] has shown that virgin PVB can reduce the coefficient of friction and wear rate of reinforced and unreinforced phenolic resins due to a protective layer formed by the PVB that mitigates the wear of the composite. With this in mind, a previous study [14] was carried out to analyze the effect that adding rPVB on a PAGF matrix has on its tribological performance during pin-on-disc wear tests. Results showed that the addition of rPVB reduced the coefficient of friction and mass loss up to 13% and 50%, respectively, an indication that rPVB might be acting as a solid lubricant. However, it has been reported that evaluating the tribological behavior of polymers is dependent on the specific test method [15] and wear mode [16] and that solid lubricants can either enhance or degrade the tribological properties of polymer matrices depending, among other factors, on tests conditions [11]; therefore, it becomes necessary to broaden the investigation regarding the use of rPVB as a solid lubricant to other wear test methods. The aim of this study is to investigate the effect of adding rPVB to a PAGF matrix on its coefficient of friction and specific wear rate during reciprocating sliding wear conditions, and to elucidate the controlling wear mechanisms through the analysis of the wear tracks and counterpart surfaces. These results can be used to extend the useful life of PAGF components by using rPVB as solid lubricant, and help reducing energy losses due to friction and wear.

## 2. Materials and Methods

### 2.1. Materials

Polymer blends were produced using a commercial glass–fiber reinforced polyamide 6 (PAGF) Ultramid B3ZG6 from BASF Co. Ltd. (Ludwigshafen, Germany) and recycled polyvinyl butyral (rPVB) donated by Reypark-Mexico (Monterrey, Mexico). Maleic anhydride (MA) with a 98.06 molecular weight from Meyer (Mexico City, Mexico) was used as a coupling agent.

### 2.2. Preparation of PAGF/rPVB/MA Blends and Specimens

**Figure 1:**
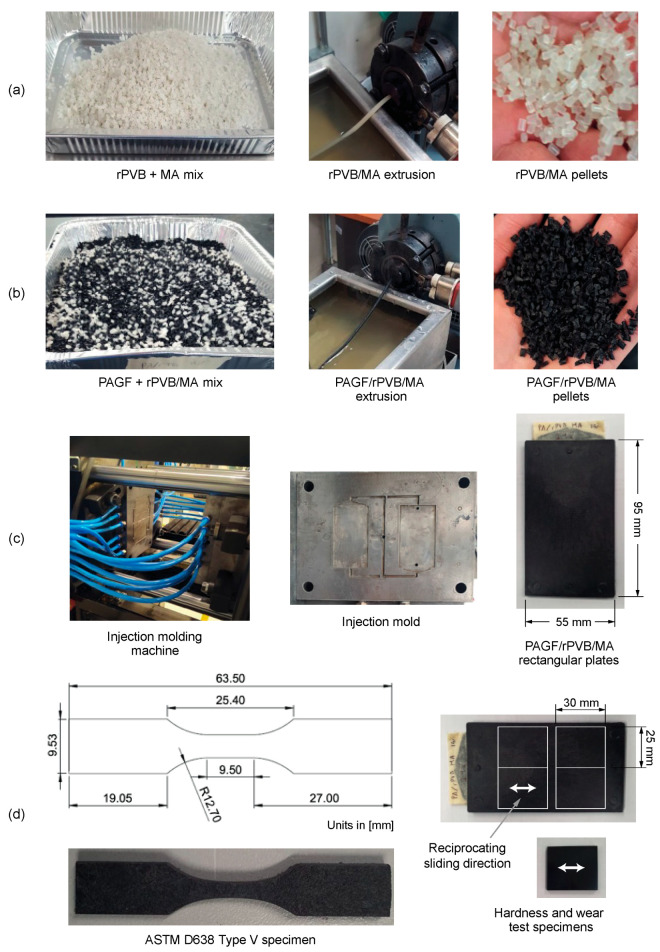
Extrusion of (**a**) rPVB/MA and (**b**) PAGF/rPVB/MA blends, (**c**) injection molding of rectangular plates, and (**d**) specimens for tensile, hardness, and wear tests.

First, a blend consisting of rPVB/MA at a 20,000:1 weight ratio was prepared in a Beutelspacher SB-19 single screw extruder at 155 °C (Figure 1a). The extruded material strand was cooled in a water bath and pelletized. Prior to mixing, both rPVB and MA were dried in a fan oven at 60 °C for 24 h. Then, PAGF/rPVB/MA blends were prepared by incorporating 10, 15, and 20 wt.% of the rPVB/MA blend into the PAGF matrix (Figure 1b). Blends were designated as 10 PVB, 15 PVB, and 20 PVB, respectively. These blends were prepared in the same extruder at temperatures between 220 and 250 °C, and again, both the rPVB/MA blend and PAGF were dried in a fan oven at 60 °C for 24 h before mixing. The obtained blends were extruded one more time to promote a good dispersion of the rPVB into the PAGF matrix. To keep an equal thermal history, the same extrusion process was performed on the PAGF matrix (0 PVB) used as control.

Rectangular plates of 95 mm × 55 mm × 3 mm of each blend were injection molded in a Belken SSF500-k5 machine (Figure 1c). Injection temperatures of 255 and 260 °C, injection pressures of 110 and 100 MPa, and packing pressures of 88 and 80 MPa were used for blends with and without rPVB, respectively. The mold temperature was 80 °C for all blends.

### 2.3. Characterization

#### 2.3.1. Etching for Morphological Analysis

Injection molded plates were cryogenically fractured in liquid nitrogen and etched with ethanol for 9 h, as reported in [9,17], to remove the rPVB from the PAGF matrix and analyze its dispersion and morphology by Scanning Electron Microscopy (SEM).

#### 2.3.2. Tensile Tests

Tensile dumbbell specimens were laser cut from the injection molded plates and tested at room temperature in a Shimadzu AG-I equipment according to ASTM D638 standard type V specimen (Figure 1d). Tests were performed at a constant crosshead displacement rate of 1 mm/min using self-aligning serrated grips. Four replicate samples per blend were used.

#### 2.3.3. Shore D Hardness Test

Shore D hardness was measured with a desktop SI-TV instrument using a 4 kg mass according to ASTM D2240 standard. Hardness tests were performed on the specimens used for wear tests (Figure 1d). Two replicate samples per blend were used, performing at least nine indentations per sample.

#### 2.3.4. Wear Tests

**Figure 2:**
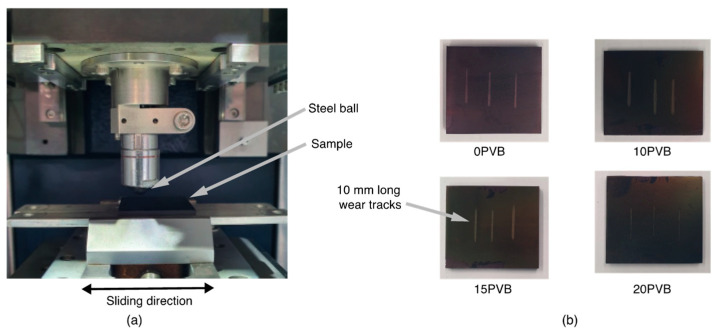
(**a**) Reciprocating sliding wear test configuration and (**b**) PAGF/rPVB specimens after wear tests (samples were sputter-coated for SEM analysis).

Ball-on-flat linear reciprocating wear tests were performed on a Bruker/UMT-2 (Bruker, Billerica, MA, USA) universal tester according to the ASTM G133 standard (Figure 2). The 25 mm × 20 mm × 3 mm rectangular specimens were saw-cut from the injection molded plates (Figure 1d) and sanded until obtaining a surface roughness of 0.13 ± 0.01 μm. A 6 mm AISI 52100 steel ball was used as a counterpart with a normal load of 10 N, a stroke length of 10 mm, a sliding speed of 30 mm/s, and a sliding distance of 200 m. Three tests per blend were performed. 

The coefficient of friction (CoF) was recorded directly from the equipment as the ratio between the tangential force and normal load, while penetration depth and volume of removed material were obtained across the wear tracks by optical profilometry with Bruker/Contour GT-K-3D (Bruker, Billerica, MA, USA) equipment.

#### 2.3.5. Scanning Electron Microscopy

A JEOL JSM-6390LV Scanning Electron Microscope (JEOL Ltd., Tokyo, Japan) was used to examine the morphology of the blends and the worn tracks after the wear tests. To eliminate charging, samples were sputter-coated before observation with a thin layer of Au/Pd in a Desk IV Denton Vacuum. Steel ball counterparts were also examined with an Olympus/GX-51 optical microscope (Olympus, Tokyo, Japan).

## 3. Results and Discussion

**Figure 3:**
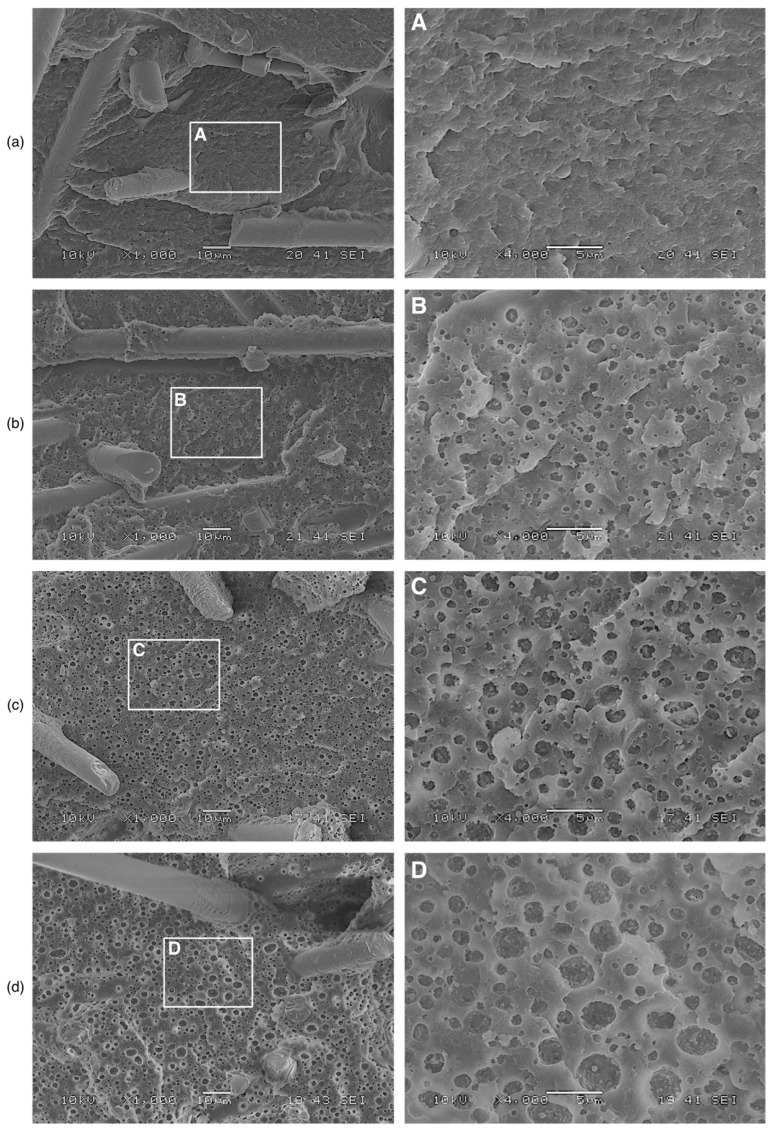
Cryogenically fractured and etched surfaces of (**a**) 0 PVB, (**b**) 10 PVB, (**c**) 15 PVB, and (**d**) 20 PVB blends.

The cryogenically fractured and etched surfaces of the PAGF/rPVB blends are shown in Figure 3 as observed by SEM. The removal of rPVB from the etched surfaces of the 10 PVB, 15 PVB, and 20 PVB is evident by the voids it left in the PAGF matrix. In general, a homogeneous dispersion of circular voids is observed, which indicates that rPVB was uniformly distributed on the PAGF matrix during extrusion and injection molding. It is also observed that the voids, thus rPVB particles, become larger with the increase in rPVB content. The image processing software ImageJ was used to measure the particle diameters and estimate the average diameter (D). Results are summarized in Table 1. As noticed before, there is an increase in the particle diameter with rPVB content, as has been reported previously [6,8]. Note, however, that the standard deviation is high, which means that particle size varies significantly in each sample.

Elastic modulus, tensile strength at break, and Shore D hardness values for the different PAGF/rPVB blends are summarized in Table 1. As observed, the addition of rPVB decreases the elastic modulus and tensile strength of the PAGF matrix by ~35% and ~40–46%, respectively, with no significant differences between the blends with rPVB. This agrees with the work by Valera et al. [6], in which a reduction in these two mechanical properties is also reported. This behavior is expected due to the lower modulus and strength of rPVB. Although not significant, it is interesting to note that tensile strength at the break for the 10 PVB, 15 PVB, and 20 PVB increases with rPVB content. Shore D hardness also decreases with rPVB content, following a linear trend (R^2^ = 0.99). However, the reduction (~2.7–6.5%) is not as significant as it is for the other properties.

**Figure 4:**
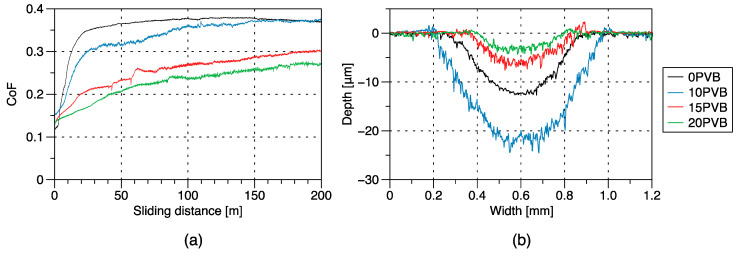
(**a**) Coefficient of friction and (**b**) cross-sectional views of the wear tracks for PAGF/rPVB blends.

The average CoF curves of the PAGF/rPVB blends are shown in Figure 4a. Two different CoF curves are observed. First, for the 0 PVB and 10 PVB blends, an initial S-shaped curve is seen, in which there is a steep increase in CoF during the first 10 m, followed by a gradual increase in CoF until the running-in period finishes, and a steady-state is reached. During the running-in period, asperities may be knocked off, the surface may mate better, and initial surface films may be formed or worn [18]. In the 0 PVB specimen, the running-in period spans up to ~60 m, after which its CoF reaches a value of 0.37, whereas, for the 10 PVB specimen, the running-in period lasts ~150 m, after which its CoF reaches the same levels of the 0 PVB specimen. Note that the increase in CoF after the S-shaped curve is less pronounced in the 0 PVB specimen. According to [11], a decrease in the duration of the running-in stage, as seen for the 0 PVB blend, can be related to the quick formation of a stable transfer film, while for the 10 PVB blend, the increase in CoF after the S-shaped curve might indicate the constant removal of the transfer film from the steel ball, which may also increase its wear rate [19]. Second, for the 20 PVB blend, CoF increases continuously, reaching a value of 0.27 at ~170 m, where it seems to stabilize. Interestingly, the 15 PVB blend shows both behaviors with an initial steep increase in CoF, similar to the S-shaped curve of the 0 PVB and 10 PVB blends, followed by a continuous increase in CoF similar to that of the 20 PVB blend, reaching a CoF value of 0.30. These results indicate that, at certain weight content, rPVB might be acting as a solid lubricant for the PAGF matrix, reducing its CoF. In Table 2, CoF mean values and standard deviation for each blend are summarized. These values were estimated from the average CoF values at the steady-state region of each sample (three per blend).

It is well documented [11,20] that for the sliding wear of polymers against metallic counterparts, the friction component resulting from adhesion equals the product of the real contact area and the shear stress of the softer material. Therefore, the friction coefficient of the polymer will be determined by these two competitive aspects. First, the contact area during sliding is determined by the hardness of the polymer, which, as mentioned previously, is slightly reduced (~2.7–6.5%) with increasing rPVB content and, therefore, might result in a slightly higher CoF when rPVB is added to the PAGF matrix. Alternatively, the shear strength of the blend significantly decreases with rPVB content (shear strength ≈ 0.5 or 0.6 tensile strength), so a final reduction in CoF is expected as the decrease in shear strength prevails over the decrease in hardness. This agrees with the 19% and 27% CoF reduction found for the 15 PVB and 20 PVB blends, respectively. For the 10 PVB case, CoF remains the same as for the 0 PVB blend, which might be related to the dominating friction and wear mechanisms during testing and will be analyzed later.

In Figure 4b, the average cross-sectional views of the wear tracks are shown, and in Table 2, the depth of the wear tracks and wear volume is summarized. The 0 PVB wear track has a depth of ~12.8 μm, which increased to ~24.6 μm for the 10 PVB blend, indicating a higher material removal when 10 wt.% of rPVB is added to the PAGF matrix. However, for the 15 PVB and 20 PVB blends, the tracks are shallower, with depths of ~7.8 μm and ~4.4 μm, respectively. Specific wear rates were estimated with the wear volume using Archard’s expression and summarized in Table 2. As expected from the wear tracks dimensions, the 15 PVB and 20 PVB blends have the lowest wear rates, 0.67 × 10^−5^ mm^3^/Nm and 0.36 × 10^−5^ mm^3^/Nm, respectively, which are 1.8 and 3.3 times lower than the 0 PVB wear rate of 1.2 × 10^−5^ mm^3^/Nm. On the contrary, the 10 PVB blend has the highest wear rate (2.2 × 10^−5^ mm^3^/Nm), which is 1.8 times higher than that of the 0 PVB blend. Again, this indicates that there might be a certain amount of rPVB in which it starts acting as a solid lubricant and decreasing the wear rate of the PAGF.

Similar results were reported by Verma et al. [12] for woven roving phenolic composites, in which the CoF and specific wear rate, obtained under pin-on-disc conditions at different sliding speeds, decreased by half with the addition of PVB containing 10 wt.% of butyraldehyde; concluding that the modification of phenolic resin by PVB not only improves its impact resistance, but also its wear resistance. It is important to mention that PVB content was kept constant in their study. Carmona et al. [14] reported a reduction between 9 and 13% for CoF and between 38 and 50% for the mass loss under pin-on-disc wear testing conditions when 10–20 wt.% of rPVB was added to a PAGF matrix. Note that in [14], wear resistance was improved at all rPVB contents, contrary to the present study in which the addition of 10 wt.% of rPVB resulted detrimental CoF and specific wear rate. This difference can be attributed to the different wear test configurations: pin-on-disc and reciprocating sliding.

Ward [21] compared the wear rates of low-carbon steel under similar conditions during continuous (pin-on-disc) and reciprocating sliding, obtaining higher wear rates for reciprocating conditions. He concluded that the difference in wear could be explained in terms of the greater amount of abrasion by the loose wear debris in the reciprocating process, in which debris is not removed as fast from the rubbing area as it is for continuous sliding. During pin-on-disc tests, some of the debris is removed from the wear track by centrifugal force, which increases with sliding speed. At high speeds, most debris will be thrown off, and only the strongly adhered debris will remain on the track. Note that pin-on-disc tests reported in [14] were performed at 0.5 m/s, whereas reciprocating sliding tests in the present study were performed at 0.03 m/s. It is possible that the mechanisms dominating the enhancement of tribological performance on the 15 PVB and 20 PVB blends are related to a decrease in debris formation, and thus the enhancement is found either on reciprocating or continuous sliding. On the contrary, for the 10 PVB blend, it is possible that debris formation was not restricted, and thus, wear under reciprocating conditions is much more severe than under continuous sliding.

**Figure 5:**
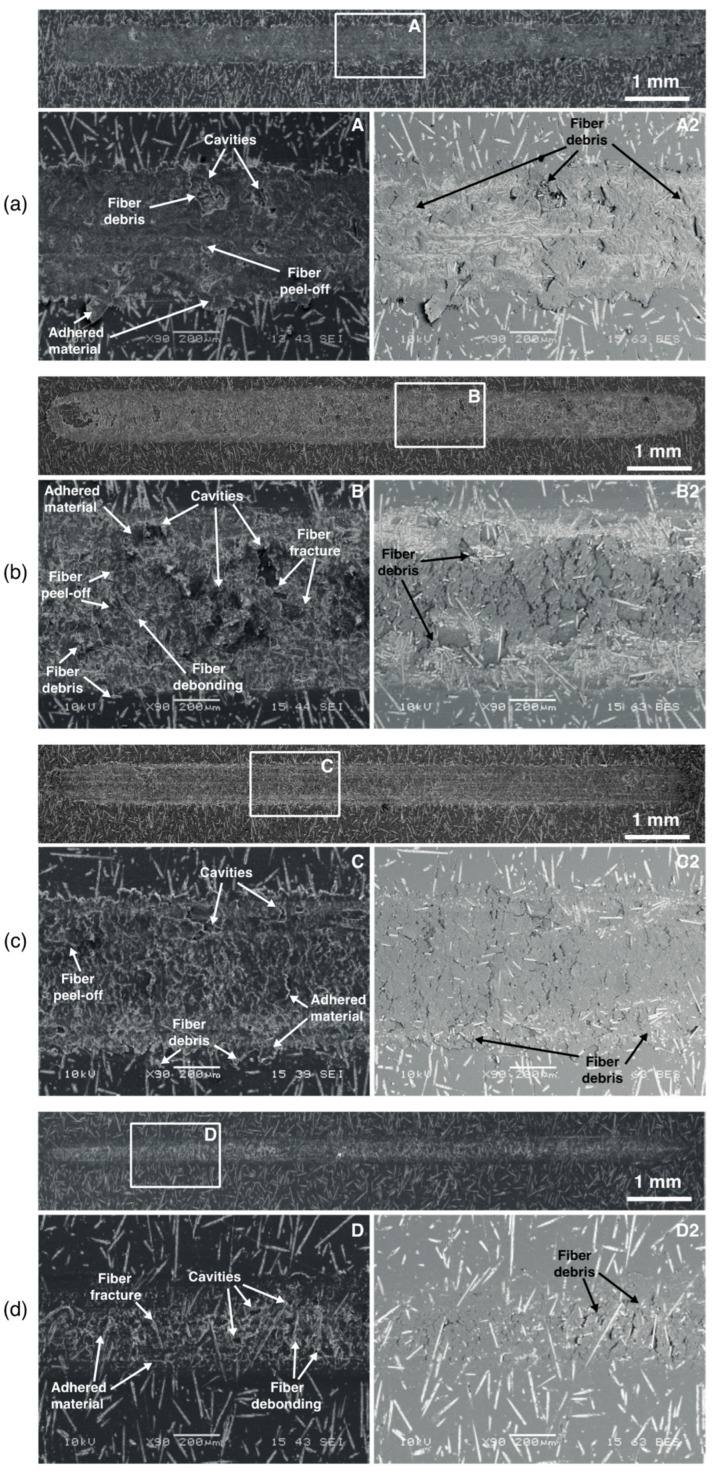
SEM micrographs of (**a**) 0 PVB, (**b**) 10 PVB, (**c**) 15 PVB, and (**d**) 20 PVB wear tracks.

**Figure 6:**
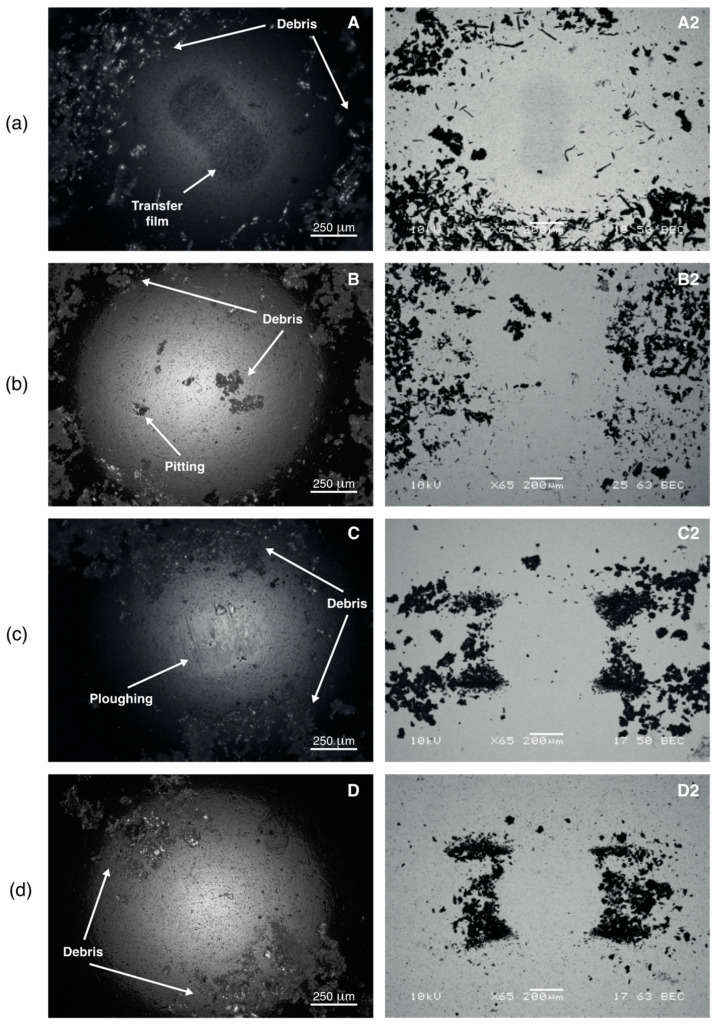
Optical and SEM micrographs of (**a**) 0 PVB, (**b**) 10 PVB, (**c**) 15 PVB, and (**d**) 20 PVB steel balls.

To understand the mechanisms behind the tribological performance of the PAGF/rPVB blends, the wear tracks (Figure 5) and steel balls (Figure 6) were analyzed by SEM. From Figure 5, it is evident that the 20 PVB specimen has the thinnest wear track, whereas the 10 PVB has the widest one, as was observed in Figure 4b. Adhesion seems to be the main wear mechanism in all the blends, followed by abrasion. In the 0 PVB blend (Figure 5a), a layer of adhered material covers the wear track. The layer has a smooth appearance and seems to have been flattened by the continuous sliding of the ball. Some of this material has been removed in certain areas generating cavities. Fiber debris is observed within these cavities, indicating that the adhered layer of material has embedded fibers in it. This was confirmed by using backscattered electrons (A2) in which the embedded fiber debris is shown in the wear track, and their alignment along the sliding direction is noticed. Some long fibers are observed, although most have been fractured and reduced to debris. This debris stuck together and formed the uniform film on the wear track.

In Figure 6a, the steel ball used during the testing of the 0 PVB blend is shown. A transfer film (adhered material) is observed at the center of the ball. This film is formed by the transfer of the material removed from the wear track to the ball. Previous studies [22] have reported that reinforcing PA with fibers reduces its specific wear rate due to the formation of a stable transfer film on the counterface. Some plowing traces are observed at the center of the ball, mainly over the transfer film area. These abrasion marks can help the interlocking and adhesion of the transferred material to the ball [23]. Lots of debris are observed at the sides, outlining the contact zone. Backscattered electrons (A2) help to distinguish the size and arrangement of the debris, which in this case (0 PVB) is formed by some long and coarse fibers and relatively large agglomerations of fibers and polymer matrix.

For the 10 PVB blend, a more destroyed worn surface was found (Figure 5b), in which a thinner and rougher layer of adhered material is also observed. Fiber fracture, peel-off, and pull-out are evident, as well as a considerable amount of fiber debris. As for the 0 PVB blend, the adhered layer has been removed in certain regions generating cavities. When analyzed by backscattered electrons (B2), a lot of fiber debris is observed; however, most of it has been evacuated to the wear track border. This is, the fiber debris is either exposed on the wear track or embedded in the adhered layer at the sides of the track. The exposed fiber debris will act as large abrasives, increasing wear rate [11] and preventing the formation, by its constant removal, of a stable transfer film on the counterpart [12]. This agrees with the higher wear volume and rate for the 10 PVB blend (Table 2). It also confirms that in the 10 PVB blend, debris formation was not reduced by the addition of rPVB, which results in higher wear rates when comparing reciprocating sliding and pin-on-disc conditions.

In the 10 PVB ball (Figure 6b), smaller plowing traces are observed at the center. However, a more severe kind of abrasion is observed: pitting. There is no evidence of a transfer film. Although the debris in this ball did not outline the contour of the contact zone as clearly as in the other blends, a larger contact area can be seen. The debris is larger and coarser than those of the 15 PVB and 20 PVB balls and is not only concentrated at the borders but also at the center. These observations agree with the exposed fibers observed in Figure 4b responsible for the severe abrasion (pitting) and the constant removal of any incipient transfer film [24].

For the 15 PVB wear track (Figure 5c), a rough adhered material layer is observed; however, fibers are not exposed as they were on the 10 PVB track. Some loose fibers or debris are observed at the borders of the track. In C2, very few fibers are observed within the adhered material layer, which might indicate that the adhered layer protected the fibers from damage, acting as a lubricant film, and that the fibers that were damaged (debris) were evacuated to the borders resulting in an almost debris-free wear track. As suggested by [11], the lower friction coefficient can be related to the reduction of fiber fracture, which results in less plowing as there are fewer rigid fiber ends in the contact surface. Some cavities are also observed; however, they are smaller, which might be a result of the increased adhesion due to the higher rPVB content. For the 15 PVB ball (Figure 6c), a reduction in the contact area is observed, as well as a lower amount of debris, which is finer (C2). Some plowing traces are observed, and no transfer film is evident.

In the 20 PVB wear track (Figure 5d), a very thin layer of adhered material seems to be covering or protecting the fibers beneath it. Some fiber damage is observed, but in general, most of the fibers are intact. D2 reveals some fiber debris that has not been evacuated to the sides. According to [12], the addition of PVB imparts ductility and a softening tendency to the polymer matrix, reducing the amount of peel-off and pull-out of fibers, which keeps the matrix and the fibers almost intact, as can be seen in Figure 5d. This agrees with the shallow wear track and low specific wear rate for the 20 PVB blend. The 20 PVB ball (Figure 6d) is similar to the 15 PVB one, with the smallest contact area and the finest debris, which agrees with the much lower amount of fiber damage in this blend as observed in its wear track (Figure 5d). Almost imperceptible abrasion marks can be seen in the center of the 20 PVB ball. As suggested by Kandanur et al. [25] in a PTFE-graphene system, it seems as if in the 15 PVB and 20 PVB blends, the rPVB might be reducing wear by regulating the debris size.

From the analysis of the wear tracks and steel balls, the wear mechanisms in each blend can be explained. For the 0 PVB blend, a stable transfer film is formed. The relative movement of the ball removes PA from the track, exposing the fibers and generating damage (fiber pull-out and fracture), which is manifested on the CoF curve by a sudden increase. Fiber debris then mixes with the PA, generating a layer of adhered material on the wear track that, with the continuous movement of the ball, is flattened. Some of the adhered material is transferred to the ball, generating a transfer film that prevents further steel/PAGF contact. This transfer film provides shielding or protection to the substrate from the damaged fibers, hindering further material removal. Previous works [22] have reported this effect on glass–fiber-reinforced polyamide.

For the 20 PVB blend, there is a preferential removal of rPVB from the PA matrix since it deforms more easily, as was reported by Byett and Allen [24] for PTFE particles in a PA66 matrix. Removed rPVB is then homogenously spread over the track, forming a thin lubricant film. There are enough large rPVB particles dispersed on the matrix (Figure 3d) to completely cover the wear track (Figure 5d). This lubricant film reduces CoF and fiber damage. A similar wear mechanism was reported by Zhang et al. [26], who reported that when a certain wt.% of graphite was added to a poly(phthalazinone ether sulfone ketone) (PPESK) resin, plenteous graphite flakes spread on the sliding surface, reducing the direct contact between the steel counterpart and the composite and therefore, reducing the friction coefficient and wear rate. Opposite from the 0 PVB blend, no stable transfer film is formed in the 20 PVB blend. As was reported for graphite and PA6 blends by Li et al. [10], the bond between the solid lubricant (in this case, rPVB) and the matrix (PAGF) may be preventing rPVB from being easily transferred onto the surface of the steel balls, hindering the formation of a transfer film on the ball.

For the 15 PVB blend, a combination of the 0 PVB and 20 PVB mechanisms is suggested. There is an initial preferential removal of rPVB from the matrix, which is spread over the track. However, this lubricant film is thinner than the one of the 20 PVB blends due to the lower rPVB content in this blend and thus provides a lower degree of protection to the fibers. Continuous sliding of the ball promotes the removal of more rPVB and PA from the matrix and exposes some fibers, promoting their damage (initial increase in CoF and plowing in the ball). However, this damage is lower than in the 0 PVB blend. The generated debris is finer and is evacuated to the sides. Verma et al. [12] concluded that the presence of PVB in a rigid phenolic resin makes the matrix more ductile, allowing more deformation and thus limiting fiber peel-off, which reduces fiber damage. Something similar might be happening in the 15 PVB blend. As for the 0 PVB blend, a film of adhered material on the wear track is formed, which consists mainly of PA and rPVB and which, by the continuous movement of the ball, is flattened, shielding the track and protecting it from further damage. However, no stable transfer film is formed, similar to the 20 PVB blend. Additionally, it is suggested that the bond between rPVB and the matrix may be preventing material transfer onto the surface of the steel balls and that if there is any incipient transfer film developing on the ball, it is removed by the few exposed fibers in the track.

For the 10 PVB blend, fiber damage cannot be prevented. On the one hand, the amount of rPVB is not enough to spread a lubricant layer on the surface of the track, which exposes the fibers from the very beginning. As for the 0 PVB blend, this generates fiber damage, which is manifested on the CoF curve by a sudden increase. Fiber debris mixes with the matrix, generating a film on the wear track composed of PA, rPVB, and fiber debris. On the other hand, no transfer film is formed (contrary to the 0 PVB blend) due to the high levels of fiber debris at the sliding surface that are likely to rub off any incipient transfer film. Additionally, once the fibers are removed from the center of the track, as observed in Figure 5b, protection of the matrix is lost, leading to more rapid material removal [24], which is even higher than the one in the 0 PVB blend. This overshadows the lubricant effect of rPVB, resulting in higher wear rates, confirming the hypothesis that there is a minimum amount of rPVB, 15 wt.% in this study, from which it starts acting as a solid lubricant.

## 4. Conclusions

The effect of adding rPVB to a PAGF matrix on its tribological performance during reciprocating sliding wear tests was investigated. It was found that adding 15 and 20 wt.% of rPVB reduces the PAGF coefficient of friction by 19% and 27%, whereas the specific wear rate was reduced by 44% and 70%, respectively. The reduction of CoF was expected as the decrease in tensile strength (40–46%), and thus shear strength, was much more significant than the decrease in hardness (2.7–6.5%) when rPVB was added to the PAGF matrix. At these wt.%, rPVB acts as a solid lubricant. However, the addition of 10 wt.% of rPVB had a detrimental effect on the PAGF tribological performance as its specific wear rate was increased by 83%, and its coefficient of friction remains unchanged, suggesting that a minimum amount of rPVB is required for it to act as a solid lubricant. This was explained by the controlling wear mechanisms that were elucidated from the analysis of the worn surfaces and steel counterparts: at high rPVB contents (15 and 20 wt.%), rPVB is spread over the surface of the wear track, forming a protective lubricant layer, which protects the fibers from damage, reducing its CoF and specific wear rates. However, at low rPVB contents (10 wt.%), fiber damage cannot be prevented as there is not enough rPVB to form a protective layer over the wear track and, opposite to the PAGF matrix, a stable transfer film cannot be formed on the counterpart as fiber damage is too great and continue rubbing-off any incipient transfer film.

## Figures and Tables

**Table 1:** The average diameter of rPVB particles in the PAGF matrix, elastic modulus, tensile strength at break, and Shore D Hardness of PAGF/rPVB blends.

Blend	Average Diameter D [μm]	Elastic Modulus [GPa]	Tensile Strength at Break [MPa]	Shore D Hardness
0 PVB	N/A	4.0 (0.3)	103.8 (10.3)	78.9 (2.3)
10 PVB	0.77 (0.26)	2.6 (0.2)	55.7 (8.1)	76.8 (2.2)
15 PVB	1.08 (0.51)	2.6 (0.2)	57.2 (4.6)	75.4 (1.8)
20 PVB	1.31 (0.68)	2.5 (0.3)	62.3 (7.4)	73.8 (2.3)

Standard deviation in parenthesis.

**Table 2:** Coefficient of friction, depth of wear track, wear volume, and specific wear rate of PAGF/rPVB blends.

Blend	CoF	Depth of Wear Track [μm]	Wear Volume [×10^−2^ mm^3^]	Specific Wear Rate [×10^−5^ mm^3^/Nm]
0 PVB	0.371 (0.001)	12.77 (0.95)	2.40 (0.10)	1.20 (0.05)
10 PVB	0.371 (0.001)	24.60 (1.47)	4.40 (0.26)	2.20 (0.13)
15 PVB	0.299 (0.001)	7.65 (0.82)	1.33 (0.06)	0.67 (0.03)
20 PVB	0.270 (0.002)	4.36 (0.70)	0.73 (0.06)	0.36 (0.03)

Standard deviation in parenthesis.

## Data Availability

The data presented in this study are available upon request from the corresponding author.
